# Acute Pazopanib-Associated Interstitial Lung Disease in a Patient With Ulcerative Colitis and Metastatic Sarcoma: A Case Report

**DOI:** 10.7759/cureus.105505

**Published:** 2026-03-19

**Authors:** Charles L Liu, Julia Kokkosis, Geremy Carpenter, Nazia Mashriqi

**Affiliations:** 1 Critical Care Medicine, Institute for Critical Care Medicine, Mount Sinai Medical Center, New York, USA; 2 Pharmacy, Mount Sinai Medical Center, New York, USA

**Keywords:** acute hypoxemic respiratory failure, blood tap pleruodesis, drug-induced interstitial lung disease (di-ild), drug induced interstitial lung diseases, pazopanib, pleomorphic sarcoma, pneumothorax pleurodesis, ulcerative colitis

## Abstract

Pazopanib is used in the treatment of advanced renal cell carcinoma and soft tissue sarcoma. While pneumothorax is a recognized pulmonary complication, interstitial lung disease (ILD) typically occurs after months of therapy. We present a rare case of acute onset ILD occurring within 72 hours of initiating pazopanib in a 43-year-old male with metastatic sarcoma and a history of ulcerative colitis. The patient developed rapidly progressive hypoxemic respiratory failure shortly after starting pazopanib. Extensive infectious and autoimmune workups were negative. Imaging findings, combined with a favorable clinical response to corticosteroids, supported the diagnosis of drug-induced ILD (DILD). The clinical course was further complicated by a persistent right-sided pneumothorax and ultimately fatal respiratory failure. This case highlights the potential for acute pazopanib-associated ILD, particularly in patients with predisposing risk factors.

## Introduction

Metastatic soft tissue sarcoma is present in approximately 10-30% of patients at the time of initial diagnosis. The prognosis remains poor in this population, with five-year survival rates ranging from 10-30% for patients presenting with metastatic disease, compared with 65-80% for those with localized disease. The lungs are the most common site of metastasis, occurring in approximately 73-80% of cases, followed by bone, lymph nodes, liver, brain, and subcutaneous tissue [1].

Pazopanib is an oral multi-targeted tyrosine kinase inhibitor approved for the treatment of advanced renal cell carcinoma and soft tissue sarcoma [2]. It exerts its antitumor effects primarily through inhibition of angiogenesis by targeting vascular endothelial growth factor receptors (VEGFR-1, VEGFR-2, and VEGFR-3), platelet-derived growth factor receptors (PDGFR-α and PDGFR-β), fibroblast growth factor receptors (FGFR-1 and FGFR-3), cytokine receptor c-KIT, interleukin-2 receptor-inducible T-cell kinase, lymphocyte-specific protein tyrosine kinase (Lck), and the macrophage colony-stimulating factor receptor (c-Fms). Pazopanib belongs to a class of targeted therapies used in metastatic soft tissue sarcoma and is commonly offered as salvage therapy in patients who experience disease progression despite standard cytotoxic chemotherapy. Other systemic treatment options in this setting include targeted agents such as regorafenib, cytotoxic agents such as trabectedin, and immune checkpoint inhibitors targeting the PD-1/PD-L1 pathway [3]. Recent studies have demonstrated that pazopanib provides meaningful clinical benefit in metastatic sarcomas, including those with pulmonary metastases [2-4].

Although pazopanib has demonstrated clinical efficacy in advanced malignancies, it is associated with several potentially serious adverse effects. Reported toxicities include hepatotoxicity characterized by elevated serum transaminases and bilirubin, QT interval prolongation with risk of torsades de pointes, fatal hemorrhagic events, and arterial thrombotic complications [5].

Pulmonary toxicities related to pazopanib have also been described. Among these, pneumothorax is the most frequently reported pulmonary complication, particularly in patients with metastatic lung lesions [5]. Proposed mechanisms include tumor necrosis with cavitation of subpleural metastases, treatment-related weakening of the lung parenchyma, and rupture of fragile metastatic nodules adjacent to the pleural surface. In addition, anti-angiogenic therapy may impair vascular repair and tissue healing, further increasing the risk of alveolar or pleural disruption [6]. Several cases of pazopanib-associated interstitial lung disease (ILD) have also been reported, typically developing after several months of therapy [7,8]. However, an acute onset of ILD shortly after treatment initiation remains rare. Here, we describe a case of rapidly progressive interstitial lung disease occurring within 72 hours of pazopanib initiation in a young patient with metastatic soft tissue sarcoma.

## Case presentation

A 43-year-old man with a history of ulcerative colitis (UC) presented to the emergency department in July 2024 with an acute onset of hypoxia and dyspnea. He was diagnosed with UC in December 2020 and initially treated with mesalamine. His course was complicated by colonic disease extension and Clostridioides difficile colitis requiring hospitalization. Therapy was subsequently escalated to infliximab 10 mg/kg every eight weeks. Due to undetectable infliximab trough levels, the dosing interval was intensified to 10 mg/kg every four weeks, resulting in clinical remission in April 2023.

In December 2019, he was diagnosed with stage T2 high-grade pleomorphic sarcoma of the right chest wall and underwent surgical resection followed by adjuvant proton radiation therapy (33 fractions over three months). In November 2022, the disease metastasized to the left upper and lower lobes, and he underwent wedge resections via video-assisted thoracoscopic surgery (VATS). His subsequent course was complicated by progressive fibrosis of the left lung, tumor invasion into the left pleural space, and development of a right lung nodule.

He later underwent repeat VATS with pneumolysis, followed by doxorubicin chemotherapy and consolidative proton radiation therapy for left pleural disease. One cycle of gemcitabine/docetaxel was administered but was complicated by diarrhea, epistaxis, fatigue, low-grade fevers, and lower extremity edema. Planned radiation therapy to the right upper lobe nodule was deferred due to the high risk of radiation-induced lung injury, given the extensive disease burden in the left lung.

**Figure 1:**
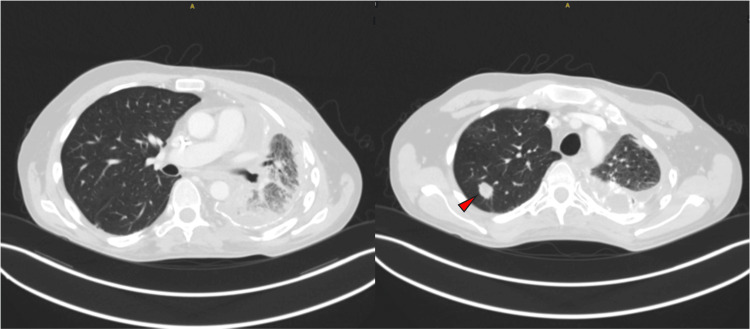
CT chest prior to pazopanib treatment showed clear right lung [A] except for a right upper lobe nodule [B, arrowhead]. Left lung consolidations and pleural scarring are stable from the previous study.

In August 2024, systemic cytotoxic therapy was transitioned to pazopanib due to disease progression. A chest CT performed before initiation demonstrated a stable fibrotic left lung and a clear right lung aside from a solitary right upper lobe nodule (Figure 1). Within 72 hours of the first dose of pazopanib, the patient developed worsening dyspnea, fever, and tachycardia, prompting presentation to the emergency department.

**Figure 2:**
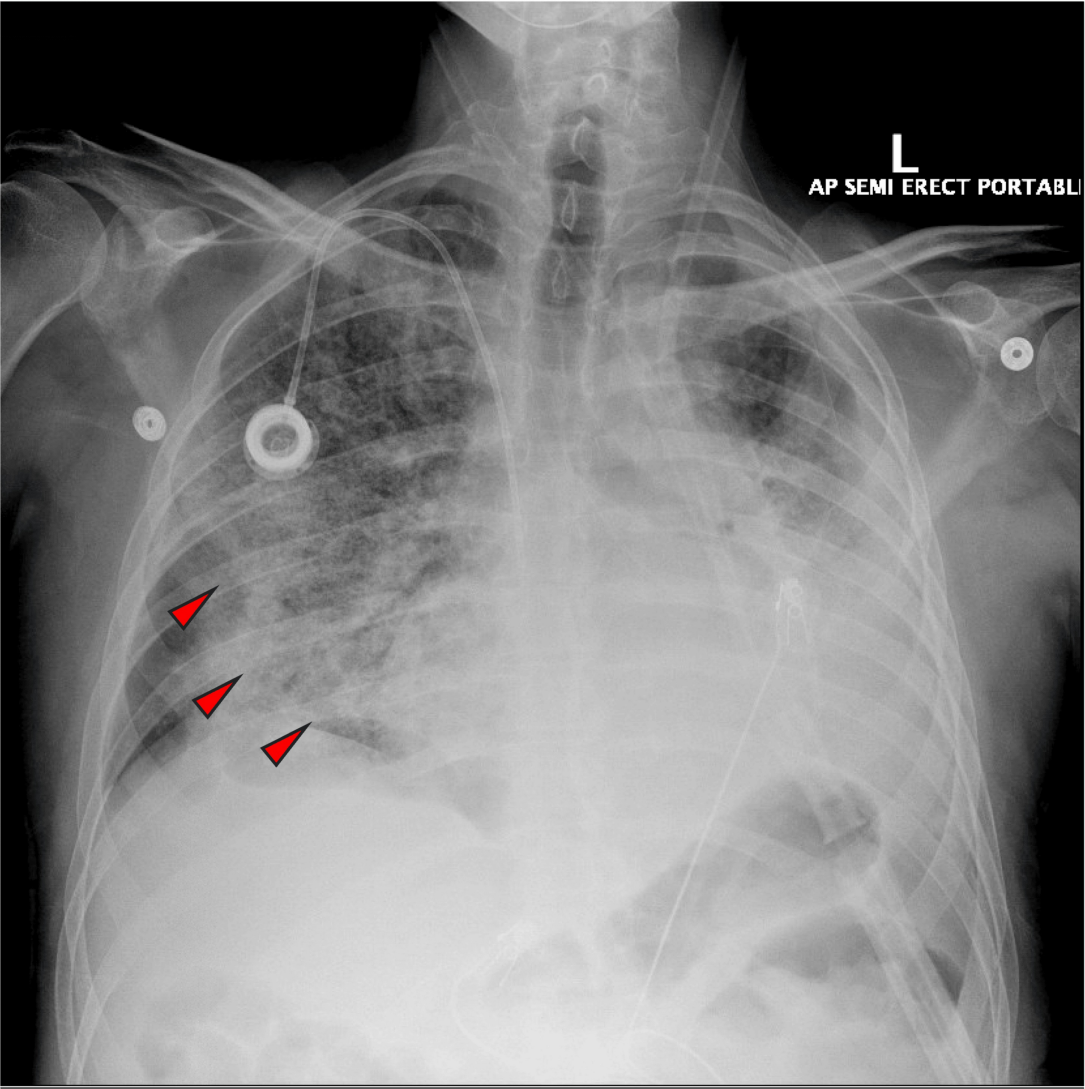
CXR showing diffuse right sided reticular infiltrates. Left chest opacities are stable from previous study.

**Figure 3:**
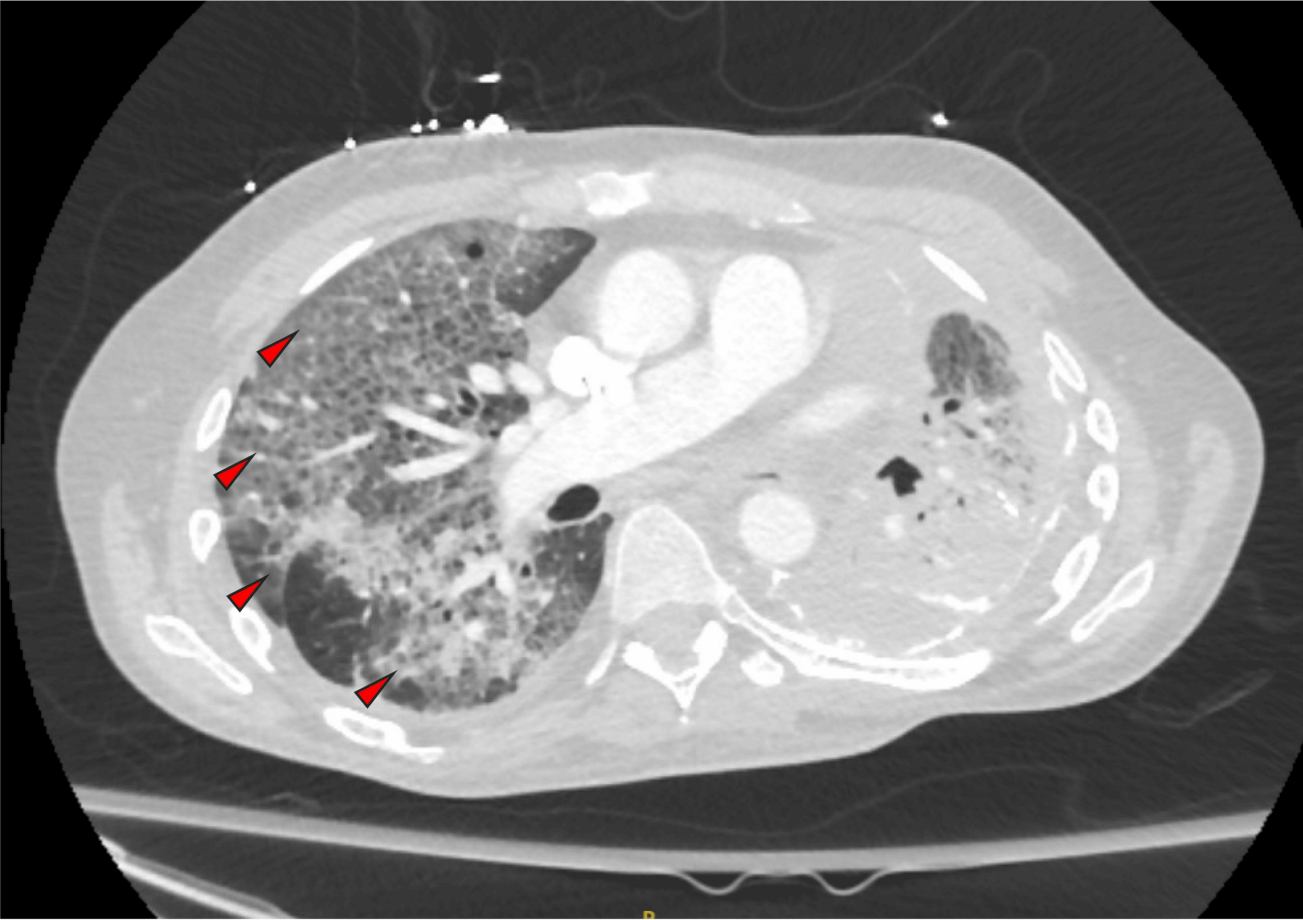
CT chest showed diffuse reticular and ground glass opacities. Left chest consolidations and pleural scarring are stable from previous study.

On arrival, the patient was profoundly hypoxic, with an oxygen saturation in the low 70s on room air. He was admitted to the intensive care unit (ICU) for acute hypoxemic respiratory failure. His temperature was 38.1 °C. Electrocardiography (ECG) demonstrated atrial fibrillation with rapid ventricular response. Lung auscultation revealed dry crackles over the right lung field and diminished breath sounds on the left, consistent with known left lung fibrosis and pleural scarring. Chest imaging demonstrated diffuse reticular and ground-glass opacities concerning for an acute interstitial process (Figures 2, 3). Laboratory evaluation revealed leukocytosis (16.7 K/µL) with markedly elevated inflammatory markers, including erythrocyte sedimentation rate (ESR) of 86 mm/hr and C-reactive protein (CRP) of 192 mg/L. Additional immunologic workup, including antinuclear antibody (ANA), antineutrophil cytoplasmic antibody (ANCA) panel, rheumatoid factor (RF), and anti-cyclic citrullinated peptide (anti-CCP) antibodies, was negative.

Blood cultures and a respiratory viral panel, including influenza and severe acute respiratory syndrome coronavirus 2 (SARS-CoV-2), were negative (Tables 1, 2). The patient was empirically started on vancomycin and piperacillin-tazobactam. Noninvasive positive pressure ventilation was initiated due to significant respiratory distress. Given the high clinical suspicion for drug-induced interstitial lung disease (DILD), high-dose methylprednisolone (1 g intravenously daily for three days) was initiated, followed by a gradual steroid taper.

**Table 1:** Blood tests on admission WBC: white blood cells, RBC: red blood cells, BUN: blood urea nitrogen, AST: aspartate aminotransferase, ALT: alanine aminotransferase, ESR: erythrocyte sedimentation rate, CRP: C-reactive protein, ANA: antinuclear antibody, C-ANCA: cytoplasmic antineutrophil cytoplasmic antibody, P-ANCA: perinuclear antineutrophil cytoplasmic antibody, RF: rheumatoid factor, Ant-CCP: anti-cyclic citrullinated peptide antibody.

Serum	Patient	Reference Range
WBC (K/uL)	16.7	4.5-11.0
RBC (M/uL)	3.42	4.50-6.00
Hemoglobin (g/dL)	9.7	13.9-16.3
Hematocrit (%)	31.5	42.0-52.0
Platelet (K/uL)	540	150-450
Sodium (mmol/L)	136	135-145
Potassium (mmol/L)	4.1	3.5-5.2
Chloride (mmol/L)	101	96-108
BUN (mg/dL)	10	6-23
Creatinine (mg/dL)	0.42	0.70-1.30
Calcium (mg/dL)	8.6	8.5-10.5
Total Protein (g/dL)	5.2	6.0-8.3
Albumin (g/dL)	2.4	3.5-4.9
AST (U/L)	27	1-35
ALT (U/L)	15	1-45
Alk phos (U/L)	85	38-126
Total Bilirubin	0.7	0.1-1.2
Glucose (mg/dL)	91	60-100
ESR (mm/h)	86	0-15
CRP (mg/dL)	192	0.0-5.0
Immunological work-up		
ANA	<1:80	<1:80
C-ANCA	<1:20	<1:20
P-ANCA	<1:20	<1:20
RF	<13	<13
Anti-CCP	4	<20

**Table 2:** Infectious workup on admission AG-DFA: antigen- direct fluorescent antibody, PCR: polymerase chain reaction, AFB: acid-fast bacilli, SARS-CoV-2: severe acute respiratory syndrome coronavirus 2, RSV: respiratory syncytial virus, CMV DNA: cytomegalovirus deoxyribonucleic acid, EIA: enzyme immunoassay.

Test	Patient	Reference Range
Sputum		
Sputum Culture	Negative	Negative
Pneumocytosis carnii AG – DFA	Negative	Negative
Pneumocytosis PCR	Negative	Negative
AFB	Negative	Negative
Respiratory Pathogens Panel by PCR		
SARS-COV-2 PCR	Not Detected	Not Detected
Influenza A PCR	Not Detected	Not Detected
Influenza B PCR	Not Detected	Not Detected
Adenovirus PCR	Not Detected	Not Detected
Parainfluenza PCR	Not Detected	Not Detected
RSV PCR	Not Detected	Not Detected
Rhinovirus PCR	Not Detected	Not Detected
Bordetella pertussis PCR	Not Detected	Not Detected
Bordetella parapertussis PCR	Not Detected	Not Detected
Chlamydia pneumoniae	Not Detected	Not Detected
Mycoplasma pneumoniae PCR	Not Detected	Not Detected
Seasonal coronavirus PCR	Not Detected	Not Detected
Blood		
Funigitell (pg/ml)	<31.00	<60.00
CMV DNA PCR	Not Detected	Not Detected
HIV-1/HIV-2 Combo Antigen/Antibody	Non-Reactive	Non-Reactive
Aspergillus galactomannan EIA	0.03	<0.5
Blood Culture	Negative	Negative
Urine		
Legionella Pneumophilia Antigen	Negative	Negative

**Figure 4:**
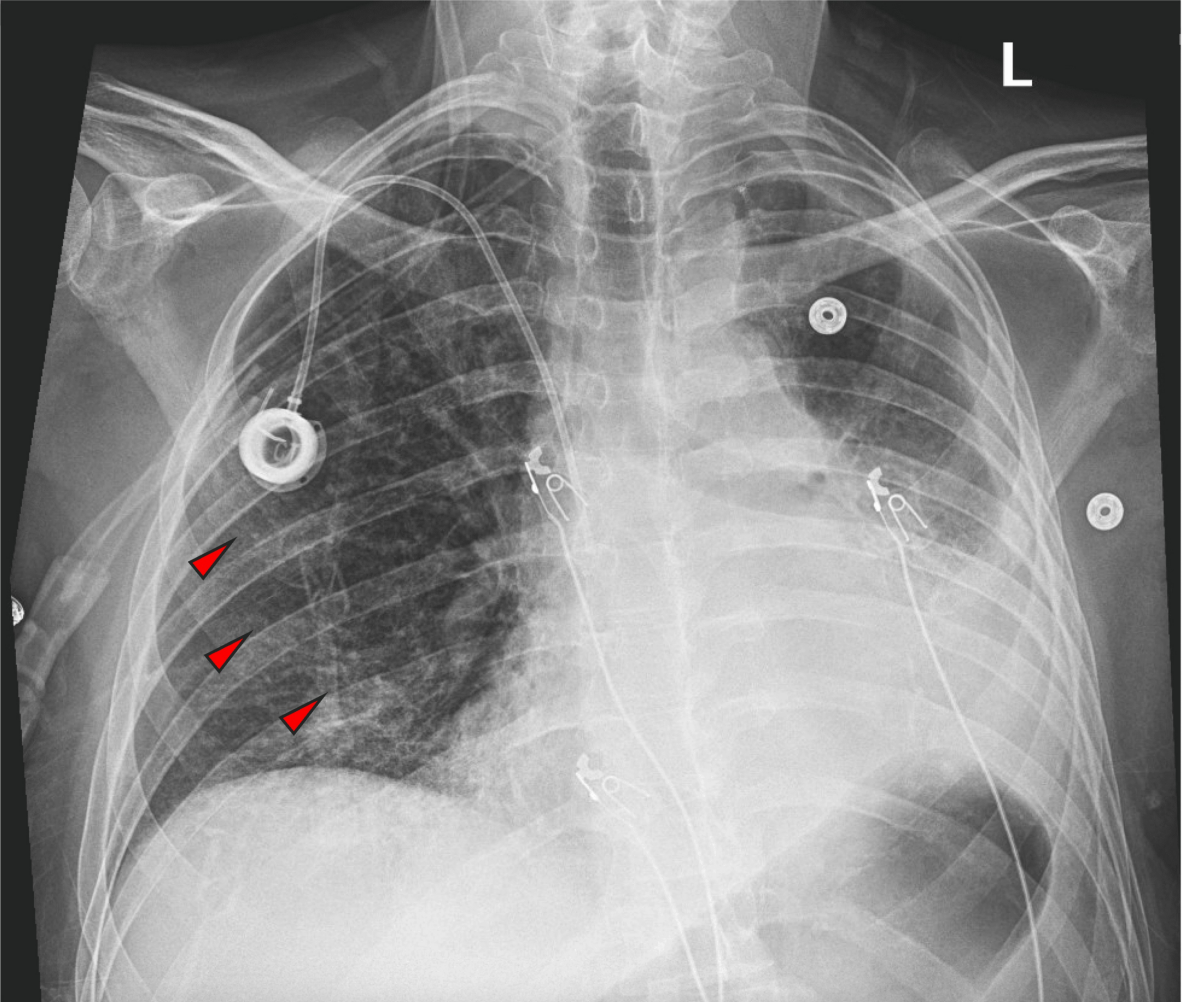
CXR showing resolution of lung infiltrates on hospital day 3.

By hospital day three, his oxygen requirement had decreased. Chest radiography (CXR) demonstrated improvement in the right-sided infiltrates (Figure 4). The patient’s clinical condition continued to improve over the following days. By hospital day 12, he was successfully liberated from a high-flow nasal cannula. His inflammatory markers also improved, with ESR decreasing to 7 mm/hr and CRP decreasing to 20 mg/L. He was subsequently transferred to the general medical floor to continue a corticosteroid taper, receiving methylprednisolone 40 mg IV daily at that time. Vancomycin was completed on hospital day 10, and piperacillin-tazobactam was completed on hospital day 25.

**Figure 5:**
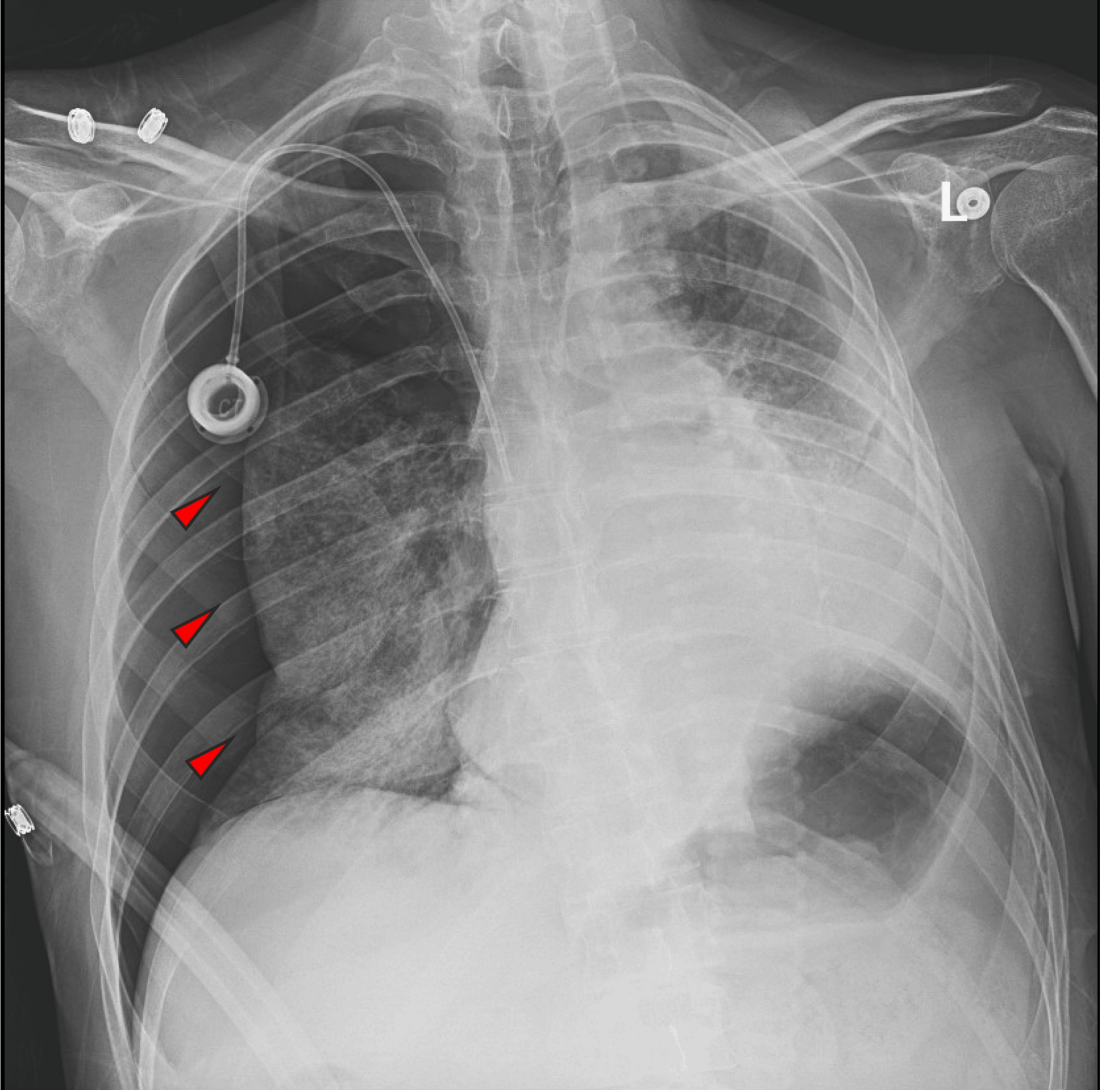
CXR showing large right sided pneumothorax.

On hospital day 13, the patient developed a right-sided pneumothorax requiring placement of a 14-Fr pigtail catheter (Figure 5). Despite continuous suction, corticosteroid tapering to prednisone 30 mg PO daily, and subsequent insertion of a 28-french surgical chest tube, a persistent air leak was observed on hospital day 30. He was transferred to a tertiary care center for further management. A talc-pleurodesis was attempted without success on hospital day 41. A subsequent blood-tap pleurodesis on hospital day 43 failed to close the persistent bronchopleural fistula.

**Figure 6:**
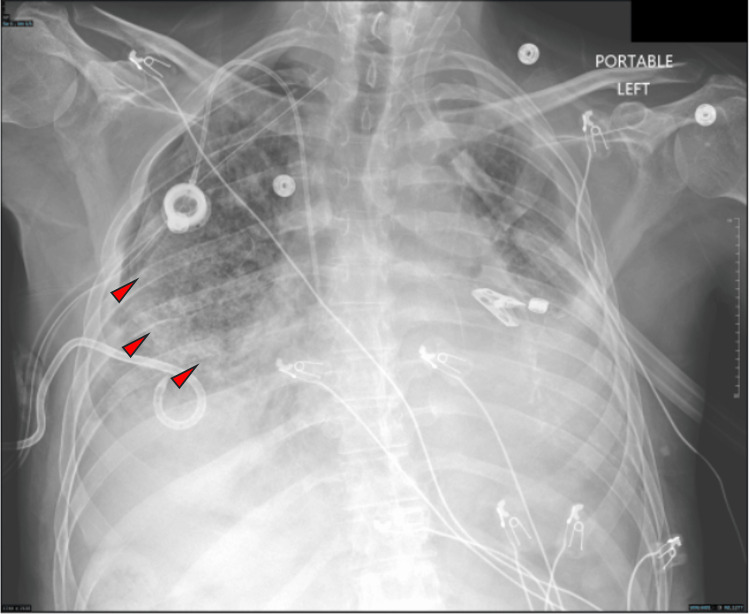
CXR showing worsening lung infiltrates

Over the following days, the patient’s respiratory status deteriorated. Imaging demonstrated worsening right-sided infiltrates (Figure 6). A sputum culture obtained on hospital day 43 grew methicillin-resistant Staphylococcus aureus (MRSA), and the patient was started on vancomycin and piperacillin-tazobactam. Placement of an endobronchial valve was considered; however, the patient was deemed medically unstable to undergo the procedure. Despite aggressive treatment, the patient’s condition continued to decline, and he passed away on hospital day 48.

## Discussion

We describe a case of acute-onset ILD occurring within 72 hours of pazopanib therapy, with hospital course complicated by persistent pneumothorax and ultimately fatal respiratory failure.

The pathogenesis of VEGFR/PDGFR TKI-associated DILD is not fully understood. VEGF inhibition by pazopanib may impair pulmonary angiogenesis, disrupt alveolar-capillary integrity, and trigger interstitial inflammation, ultimately contributing to ILD development [8].

Previous studies suggest that patients with pre-existing interstitial lung disease (ILD) may be more susceptible to tyrosine kinase inhibitor (TKI)-induced lung toxicity. This phenomenon has also been observed with other VEGFR-targeting TKIs, including sorafenib and sunitinib [9], potentially due to increased vulnerability of the pulmonary interstitium to inflammatory injury in the setting of underlying structural lung disease. Although our patient did not have documented ILD before presentation, ulcerative colitis (UC) is associated with extraintestinal pulmonary manifestations, including ILD, and may predispose patients to immune-mediated lung injury [10]. In addition, observational and mechanistic data suggest that anti-tumor necrosis factor-α (anti-TNF-α) agents, including infliximab, can precipitate or exacerbate ILD, often occurring within weeks of infusion [11]. Our patient last received infliximab in April 2023, more than one year before admission, making direct infliximab-associated lung toxicity unlikely; however, prior immune-modulating therapy may have contributed to underlying pulmonary susceptibility.

Opportunistic infections could not be definitively ruled out, as bronchoscopy was not performed. However, sputum cultures and serologic testing were negative for Cytomegalovirus (CMV), $beta$-D-glucan, aspergillus antigen, acid-fast bacilli (AFB), and Pneumocystis jirovecii. A comprehensive autoimmune workup, including ANA, ANCAs, rheumatoid factor, CCP, dsDNA, anti-histone, anti-U1-Ribonucleoprotein antibodies (RNP), anti-Jo, and anti-la, was also negative.

Viral pneumonia was considered but was excluded based on a negative respiratory viral panel.

Radiation-induced interstitial lung disease was unlikely, as the patient’s most recent radiation therapy had occurred more than four years earlier. Radiation pneumonitis typically develops within six to 12 weeks of therapy, and radiation-induced fibrosis typically manifests within six to 24 months. [12,13].

The management of drug-induced interstitial lung disease (DILD) depends on the severity of respiratory compromise. Mild cases may improve with discontinuation of the offending agent alone and close clinical monitoring, whereas moderate to severe cases typically require systemic corticosteroid therapy in addition to withdrawal of the suspected drug. In patients with severe hypoxemia or rapidly progressive respiratory failure, high-dose corticosteroids are often recommended as initial treatment [14].

In this case, the patient presented with acute hypoxemic respiratory failure requiring intensive care unit admission, consistent with severe DILD. He demonstrated both clinical and radiographic improvement following treatment with high-dose corticosteroids, further supporting the diagnosis of DILD. Additionally, the subsequent development of a persistent pneumothorax, an adverse event previously reported in association with pazopanib therapy [4], further implicates the drug as the likely causative agent.

Gemcitabine/docetaxel-associated ILD is also possible, as these agents are known to cause lung toxicity [15]. However, in this case, the patient received only one cycle more than two months prior to the onset of symptoms, making direct causation less likely. It is plausible that prior gemcitabine/docetaxel exposure increased susceptibility to pazopanib-induced lung injury.

The immediate cause of death was most likely MRSA pneumonia, to which the patient was particularly susceptible due to prolonged hospitalization and extended exposure to high-dose corticosteroids. This case highlights the increased risk of opportunistic and nosocomial infections in patients undergoing treatment for drug-induced interstitial lung disease (DILD), particularly when prolonged immunosuppressive therapy is required. Clinicians should maintain a high index of suspicion for secondary infections in this setting, and clinical deterioration may warrant a low threshold for initiating empiric broad-spectrum antimicrobial therapy.

## Conclusions

Pazopanib-associated ILD, while exceedingly rare, is a serious and potentially life-threatening adverse event. This case illustrates that ILD can develop rapidly after pazopanib treatment, especially in patients at increased risk for ILD - such as those with ulcerative colitis or prior history of ILD. Early recognition of drug-induced ILD is critical: immediate discontinuation of the offending agent and prompt initiation of corticosteroid therapy can be lifesaving. Clinicians should remain vigilant for respiratory complications in patients receiving pazopanib. This includes maintaining a low threshold for chest CT and emergency department referral at sign of respiratory compromise. The broader implication for oncology practice is that even targeted therapies with generally favorable safety profiles can unmask severe idiosyncratic reactions. As the use of tyrosine kinase inhibitors expands, reporting and awareness of rare toxicities like ILD become essential. Going forward, accumulation of similar case reports will help in identifying possible predisposing factors and formulating guidelines for monitoring. Ultimately, this case underscores the need for multidisciplinary management and a high degree of clinical suspicion to ensure patient safety when managing advanced cancers with novel targeted agents.

## References

[1] Diessner BJ, Weigel BJ, Murugan P, Zhang L, Poynter JN, Spector LG (2020). Associations of socioeconomic status, public vs private insurance, and race/ethnicity with metastatic sarcoma at diagnosis. JAMA Netw Open.

[2] van der Graaf WTA, Blay JY, Chawla SP (2012). Pazopanib for metastatic soft-tissue sarcoma (PALETTE): a randomised, double-blind, placebo-controlled phase 3 trial. The Lancet.

[3] Söylemez CM, Gürsoy P, Şanli UA (2025). Response rates of pazopanib therapy in metastatic soft tissue sarcoma using real‑world data. Oncol Lett.

[4] Irimura S, Nishimoto K, Kikuta K (2015). Successful treatment with pazopanib for multiple lung metastases of inguinal epithelioid sarcoma: a case report. Case Rep Oncol.

[5] Aiba H, Kimura H, Yamada S (2021). Different patterns of pneumothorax in patients with soft tissue tumors treated with pazopanib: a case series analysis. PLoS One.

[6] Sternberg CN, Davis ID, Mardiak J (2010). Pazopanib in locally advanced or metastatic renal cell carcinoma: results of a randomized phase III trial. J Clin Oncol.

[7] Ide S, Sakamoto N, Hara S (2017). Interstitial Lung Disease Induced by Pazopanib Treatment. Intern Med.

[8] Harada Y, Kakimoto S, Shimizu T (2020). Pazopanib-associated interstitial lung disease in a patient with renal cell carcinoma. BMJ Case Rep.

[9] Horiuchi-Yamamoto Y, Gemma A, Taniguchi H (2013). Drug-induced lung injury associated with sorafenib: analysis of all-patient post-marketing surveillance in Japan. Int J Clin Oncol.

[10] Larsen S, Bendtzen K, Nielsen OH (2010). Extraintestinal manifestations of inflammatory bowel disease: epidemiology, diagnosis, and management. Ann Med.

[11] Huang Y, Lin W, Chen Z, Wang Y, Huang Y, Tu S (2019). Effect of tumor necrosis factor inhibitors on interstitial lung disease in rheumatoid arthritis: angel or demon?. Drug Des Devel Ther.

[12] Gross NJ (1977). Pulmonary effects of radiation therapy. Ann Intern Med.

[13] Giridhar P, Mallick S, Rath GK, Julka PK (2015). Radiation induced lung injury: prediction, assessment and management. Asian Pac J Cancer Prev.

[14] Poveda JJ, Tamayo MH, Huizi JJ, Sellares J, Hernández-González F (2026). Drug-induced pulmonary toxicity in the era of immunotherapy and biologics: a narrative review of mechanism, diagnosis, and management. Pulm Ther.

[15] Binder D, Hübner RH, Temmesfeld-Wollbrück B, Schlattmann P (2011). Pulmonary toxicity among cancer patients treated with a combination of docetaxel and gemcitabine: a meta-analysis of clinical trials. Cancer Chemother Pharmacol.

